# How plants cope with temperature stress

**DOI:** 10.1186/1741-7007-9-79

**Published:** 2011-11-17

**Authors:** Virginia Walbot

**Affiliations:** 1 Department of Biology MC5020, 385 Serra Mall, Stanford University, Stanford CA 94305-5020, USA

## Abstract

A cold night can follow a hot day, and because they cannot move, plants subjected to such temperature fluctuations must acclimate on the basis mainly of pre-existing proteins. Zhang *et al*. report in a paper in *BMC Plant Biology*, however, that heat-induced cell death results from transcriptional activation of a kinase related to disease resistance factors and leading to a localized hypersensitive response. This specialized response reflects the failure of adaptations that normally enable plants to survive over a remarkable temperature range, by mechanisms that are not fully understood.

## Commentary

When you camp out in the high deserts of Southern California, it freezes at night. As you huddle in your sleeping bag waiting for the coffee water to boil, the sun rises and plants begin to photosynthesize using the first photons in the cool dawn. Later that day while you are shedding clothing and gulping water in 45°C heat, the same plants conduct photosynthesis under a blazing sun. It is remarkable that plants adapted to high deserts thrive despite approximately 50°C daily temperature swings [1]. Transcription, translation, membrane properties, mitochondrial respiration, microtubule and microfilament-mediated processes, plastids, and all other essential cell functions retain activity over a broad temperature range, and furthermore, all of these processes remain in balance [2]. How can plant cells, tissues and organs sustain homeostasis despite temperature fluctuations? Of course, for temperate and tropical zone plants and crops, the fluctuations are less extreme over a typical day, but nonetheless changes of 10 to 15°C over a day or a week are readily accommodated. Only when some process fails as a result of heat or cold does the local temperature regime set the limits of plant distribution [3]. Zhang *et al. *[4] provide an example of failure, with evidence that exceeding the homeostatic limits for managing reactive oxygen species at high temperature results in localized cell death.

## Efficiency versus adaptability

**Figure 1:**
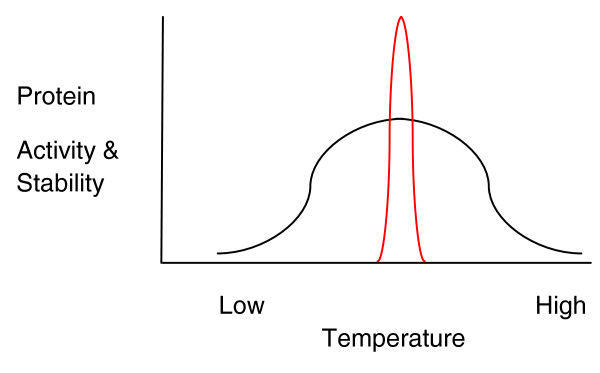
**Schematic representation of the temperature tolerance of plant and animal processes**. Plant proteins (black line) will show a broad range of activity and stability relative to temperature in comparison to mammalian proteins (red line) with a peak narrowly centered on body temperature.

At the level of individual proteins, there must be a compromise between peak efficiency at constant temperature - the route taken by mammals - and sufficiency over a broad temperature range (Figure 1). To state it another way, mammalian enzymes and constituent processes fail outside a narrow window of optimal temperatures, and such failure is catastrophic for the organism, whereas plants can maintain and coordinate cellular processes over a broad temperature range.

**Figure 2:**
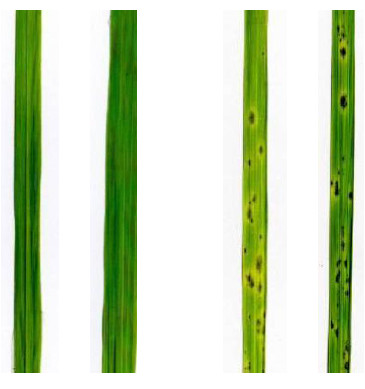
**Necrotic lesions on leaves grown at non-permissive temperature**. Rice leaves overexpressing *NRKe*, a gene encoding a kinase related to known disease-resistance kinases that induce necrosis in response to infection, are grown for 10 days at 24°C (the two blades on the left) and then for 3 days at 35°C (the two blades on the right). At 35°C, the non-permissive temperature, the leaves develop necrotic lesions. Taken from Figure 2b of Zhang *et al. *[4].

When coordination between key cellular processes fails, cell death can result. This can occur in plant cells as part of a localized hypersensitive response to pathogen attack, a strategy to restrict the growth of the invader. Similar localized cell death lesions can result from failure of homeostatic processes at the non-permissive temperature in plant mutants with specific sensitivity to either heat or cold. In both cases this reflects an inability to manage reactive oxygen and its consequences. In the case reported by Zhang *et al. *[4] high temperature is non-permissive (Figure 2). In contrast, the dominant *Lesion mimic1 *(*Les1*) maize mutant is entirely normal at high temperature but at 22°C or lower, extensive necrotic leaf lesions develop [5]. Wounding leaves with pins or painting leaves with low molecular weight organic compounds (the sort found in marking pens used to write on maize leaves) can trigger lesion symptoms at any temperature. The observations of Zhang *et al*. suggest that pathways activated in pathogen defense may also play a part in integrating responses to the abiotic challenges of substantial temperature fluctuations. It remains mysterious, however, how plants generally manage to maintain homeostasis in the face of such fluctuations.

## Is the solution to temperature fluctuation duplication or stabilization of proteins?

Varying by plant species, alleles of duplicated genes in tetraploid plants could be selected to contribute to temperature buffering; but only the most recent tetraploid possibly retains all duplicated genes, so this cannot account for adaptability in the majority of plants. If subfunctionalized loci are necessary for temperature adaptation, gene loss and allele fixation at single loci could be a recipe for disaster in a fluctuating environment, because they would result in numerous processes vulnerable to high and low temperatures. I predict instead that much of the buffering against temperature perturbation in protein complexes and in process coordination will require an extra, perhaps novel and plant-specific, suite of stabilizing proteins.

To me, the retention of fidelity in nucleic acid-based processes is the most striking. Empirical laboratory evidence amply demonstrates that interactions of proteins, such as transcription factor binding to short DNA motifs or charged tRNAs with three bases in mRNA, are stable over a very narrow temperature range. *In vivo*, chromatin structure could stabilize protein-DNA interaction to regulate transcription initiation, and the ribosome similarly provides a special niche for translation. In plants, I anticipate that proteins will be identified that stabilize specific, local chromatin configurations within the normal temperature range for a given habitat. Similarly, I predict that there will be proteins to stabilize the ribosome to permit the otherwise tenuous interactions within the molecular complexes to continue with high fidelity and efficiency. These stability factors would prevent both disassociation of factors that act together and persistence of interactions between proteins whose disassembly is required for normal regulation of cellular processes. I imagine that the various molecular complexes defined in laboratory yeast and in mammals will have accessory proteins. I predict that these will be more likely to be defined by biochemistry than genetic approaches, because under optimal conditions they may be dispensable.

## Continuous development as a strategy for organ acclimation

**Figure 3:**
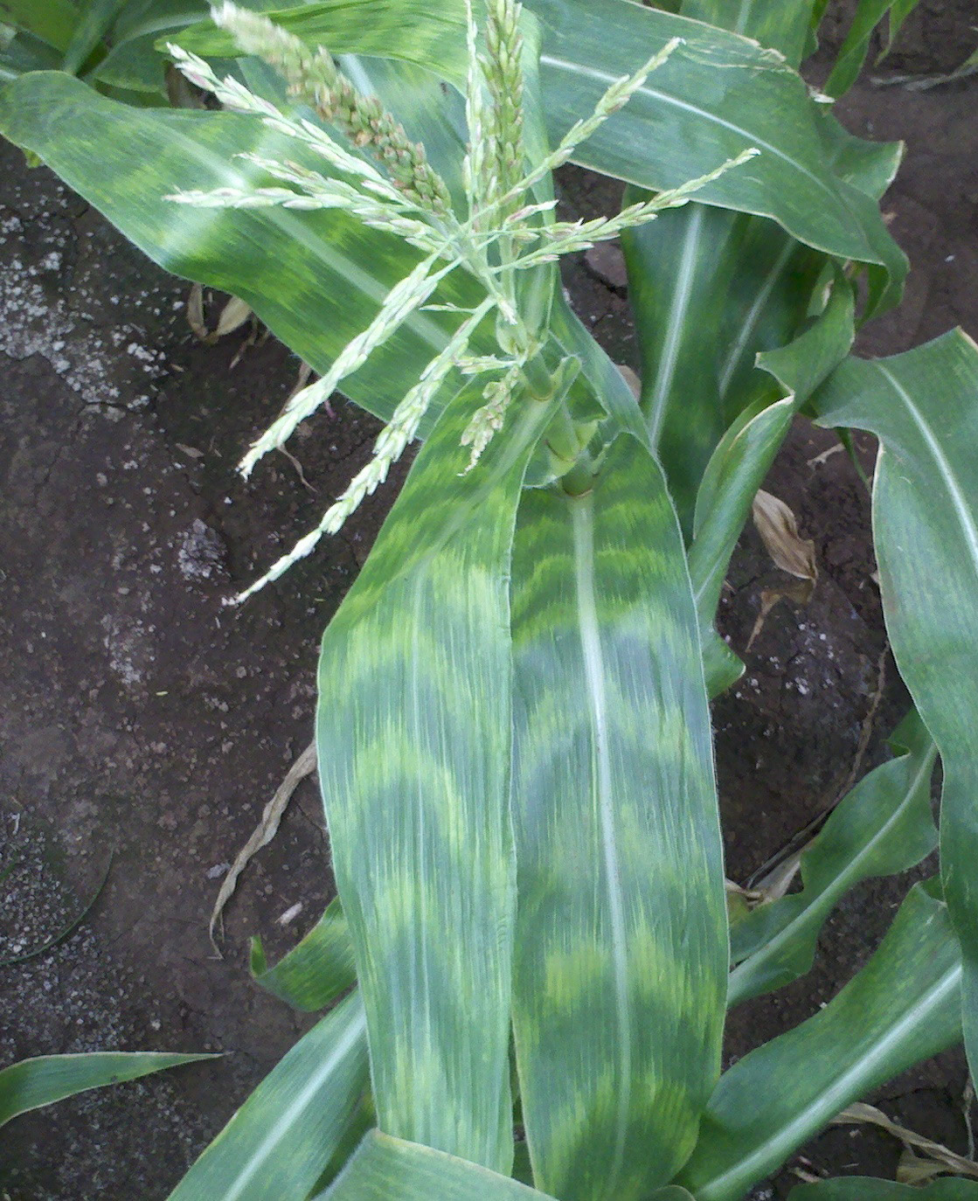
**Zebra striping in a fully mature corn plant grown on Moloka'I, Hawaii in winter 2011**. All of the adult leaves were marked by crosswise normal dark green and chlorotic (yellow green) bands. It takes about 10 days for an adult leaf to develop, and pairs of green and yellow-green bands are inferred to represent a 24-hour period of leaf development. Without further tests it is not possible to pinpoint whether normal chloroplast development or stability is heat or cold sensitive. Ambient day temperatures were 23 to 27°C, just slightly elevated over night temperatures of 17 to 21°C. Zebra striping is highly persistent in maize because zones of poor differentiation are not repaired during growth at the permissive temperature. Photograph courtesy of Tim Kelliher, Stanford University.

The plant life strategy of continuous development also contributes to maintenance of function in a fluctuating environment. Plants acclimate to current conditions by integrating environmental information with developmental programs for leaf and stem or root initiation from meristems and subsequent growth and differentiation. An example of acclimation to light conditions is exploited by the nursery industry to entice you to purchase plants. By starving plants for photons in dim light, horticulturists generate plants with larger, darker green leaves. After you have bought the plant and installed it in a sunny window, all of the luxuriant shade leaves senesce precociously and smaller, paler sun leaves emerge that are more suited to the high-light environment. Zebra-stripe mutants of maize mirror the alternation of cool dark nights with warmer bright days through failure to stabilize chlorophyll content (Figure 3) for half of each 24-hour period in response to non-permissive light or temperature conditions, with the consequence that crosswise strips of pale tissue alternate with normal green tissue throughout the 10-day period of blade development. In many grasses, defective stripes are not restored to wild-type phenotype but remain a report on temperature fluctuations beyond the optimal range over the course of leaf growth.

## Key stages when temperature tolerance really matters

Germination in flowering plants is the irreversible growth of a plant embryo out of the protective seed coat, fueled by stored nutrient reserves. As germination proceeds, there is a race between the rate of reserve consumption and the establishment of an independent, photosynthetically competent seedling able to acquire water and mineral nutrients from the soil. Germination is highly sensitive to temperature in many species. First, many species are triggered to germinate by either a high or low temperature period that destroys germination inhibitors, an adaptation allowing the plant to measure the end of winter for spring emergence or end of summer for fall germination. Second, water spurs imbibition, making growth possible, but a subsequent drop in temperature can freeze the tender seedling stem, while high heat will crisp the unfurling preformed leaves beyond repair, under conditions that can be tolerated by a well established plant.

Flowering represents another one-way commitment in the lifecycle, as an apical meristem previously generating leaves and stems switches to the floral program and is entirely consumed in making a flower. Although heat and cold can adversely affect the showy floral parts, the most serious impact is primarily on the developing haploid pollen and its nutritive diploid support tissue, the tapetum. The parallel with germination is that pollen is sealed off from the vegetative plant shortly after meiosis by a thick coat and must survive with a fixed nutrient store throughout maturation, dispersal, and the initial stages of pollen tube growth prior to fertilization. Nutrients in pollen pass through the tapetal layer and the quality of this single-cell-thick tissue ring is thus also paramount. In tomato, slight temperature elevation that did not affect plant biomass, number of flowers, or meiosis greatly affected the number of functional pollen grains and hence fruit yield [6]. In rice, low temperature limits cool season production because of the negative impact on male reproductive fitness [7]. The literature on male fitness abounds with examples of the negative impact of temperature extremes tolerated by the vegetative plant.

Considering both vegetative (leaf phenotype) cases and male sterility, it is clear that temperatures just beyond the acclimation range can greatly affect both survival and reproduction. These cases show that plants can thrive across a broad temperature range, but that temperatures beyond genotypic thresholds evoke consequences such as cell death - as demonstrated by Zhang *et al*. - poor greening, and male sterility. These deleterious phenotypes are the starting point for unraveling the mechanisms underpinning temperature tolerance, with the hypothesis that the first process to fail at either high or low temperature defines a key component of plant life.
